# Influence of *Staphylococcus aureus* on Outcomes after Valvular Surgery for Infective Endocarditis

**DOI:** 10.1186/s13019-017-0623-3

**Published:** 2017-07-20

**Authors:** Sang Myung Han, Robert A. Sorabella, Sowmya Vasan, Mark Grbic, Daniel Lambert, Rahul Prasad, Catherine Wang, Paul Kurlansky, Michael A. Borger, Rachel Gordon, Isaac George

**Affiliations:** 1 Division of Cardiothoracic Surgery, New York Presbyterian Hospital – College of Physicians and Surgeons of Columbia University, 177 Fort Washington Ave, MHB 7GN-435, New York, NY 10032 USA; 2 Division of Infectious Diseases, New York Presbyterian Hospital – College of Physicians and Surgeons of Columbia University, New York, NY USA

**Keywords:** Endocarditis, Aortic valve replacement, Heart valve

## Abstract

**Background:**

As *Staphylococcus aureus* (SA) remains one of the leading cause of infective endocarditis (IE), this study evaluates whether *S. aureus* is associated with more severe infections or worsened outcomes compared to non-*S. aureus* (NSA) organisms.

**Methods:**

All patients undergoing valve surgery for bacterial IE between 1995 and 2013 at our institution were included in this study (*n* = 323). Clinical data were retrospectively collected from the chart review. Patients were stratified according to the causative organism; SA (*n* = 85) and NSA (*n* = 238). Propensity score matched pairs (*n* = 64) of SA versus NSA were used in the analysis.

**Results:**

SA patients presented with more severe IE compared to NSA patients, with higher rates of preoperative vascular complications, preoperative septic shock, preoperative embolic events, preoperative stroke, and annular abscess. Among the matched pairs, there were no significant differences in 30-day (9.4% SA vs. 7.8% NSA, OR = 1.20, *p* = 0.76) or 1-year mortality (20.3% SA vs. 14.1% NSA, OR = 1.57, *p* = 0.35) groups, though late survival was significantly worse in SA patients. There was also no significant difference in postoperative morbidity between the two matched groups.

**Conclusions:**

SA IE is associated with a more severe clinical presentation than IE caused by other organisms. Despite the clearly increased preoperative risk, valvular surgery may benefit SA IE patients by moderating the post-operative mortality and morbidity.

## Background

Infective endocarditis (IE) remains a condition associated with high morbidity and mortality rates despite advances in surgical techniques and medical therapy [1]. *Staphylococcus aureus* (SA) is one of the leading causative organisms of IE, and has shown an increasing prevalence in recent years [2]. Among the many pathogens cultured from patients with IE, SA is particularly concerning as it may be associated with higher mortality rates, ranging from 23 to 45% in published reports [3]. In addition to increased mortality, several studies have reported that SA IE is associated with a more severe clinical presentation, including embolic phenomenon and neurologic involvement, and higher rates of persistent infection than IE caused by non-*Staphylococcus aureus* (NSA) pathogens [4].

Few studies have been specifically devoted to characterizing the virulence of SA in comparison to patients with NSA IE, and those reports that do exist are limited by a small sample size. Additionally, no study has directly investigated the distinct presenting features or postoperative outcomes associated with SA IE compared to NSA in a surgical population requiring valvular replacement. Therefore, the aim of the current study is to evaluate the clinical presentation of patients with culture-positive SA IE, and compare postoperative morbidity and mortality to patients requiring surgery for NSA IE. Characterizing these differences may help identify high-risk patients in whom early surgery may be the optimal treatment strategy.

## Methods

### Patient selection and Data collection

We conducted a retrospective analysis of all patients undergoing valve replacement surgery for IE at our institution between April 1995 and April 2013. Initially subjects 18 years and older were identified using validated ICD-9 codes for IE as the primary discharge diagnosis. This yielded approximately 1411 patients with a diagnosis of IE, of which 323 surgical patients for inclusion into the study. All patients met criteria for IE according to the modified Duke criteria. Patients were then stratified according to their causative organism: patients with SA IE (Group SA, *n* = 85) and patients with NSA IE, including culture-negative IE (Group NSA, *n* = 238). The study was approved by the Columbia University Institutional Review Board and need for individual patient consent was waived.

All data were collected from the electronic medical record. Variables included basic demographics, co-morbidities, New York Heart Association (NYHA) functional class, presenting symptoms, events preceding surgery (i.e. vascular complications, immunological phenomenon, new or worsening congenital heart failure (CHF), persistent bacteremia, leukocytosis, septic shock, embolic event, stroke, and myocardial infarction), preoperative lab values, blood culture data, echocardiographic parameters and characteristics, operative details including procedure and cardiopulmonary bypass data, postoperative complications, and discharge disposition. In-hospital mortality was obtained from the electronic medical record and survival for patients lost to follow-up was obtained from the United States Social Security Death Index.

### Statistical analysis

Analysis was performed using STATA 13.1 (Stata-Corp LP, College Station, TX, USA). All patients were included in the final analysis. Continuous variables are expressed as mean ± standard deviation and compared using independent samples t-tests. Categorical variables are presented as overall counts and percentage of the group and are compared using Pearson’s chi-square test or Fisher’s exact test where applicable. We computed propensity scores (PS) to adjust for the different baseline characteristics among the SA vs NSA patients. PS scores were estimated by building a multivariable probit model, adjusting for all the baseline demographic, clinical and echocardiographic characteristics of the patients listed in Tables 1. Propensity scores were matched with a caliper range of 0.05 to obtain the matched pairs of SA and NSA patients. Conditional logistic regression models were built among the matched pairs to assess the effect of exposure (SA vs NSA) on various outcomes. Univariable and multivariable Cox proportional hazard regression models were used to assess hazard ratios (HRs) with 95% confidence intervals (CIs) comparing mortality in SA and NSA patients. In order to identify predictive factors for mortality at 30 days, a univariable Cox proportional hazard regression was performed using clinically relevant baseline co-variables. Significant associations, defined as univariable associations with *p*-values less than 0.2, were entered into a multivariable Cox proportional hazard regression model. The association of risk predictors was assessed by Wald-statistics. Kaplan-Meier analysis was used to compare late survival, and survival curves were compared using the log-rank test. All *p* values <0.05 were considered significant, and all reported *p* values were two-sided.

**Table 1:** Baseline, Clinical, and Echocardiographic Characteristics of Patients

	Overall (*n* = 323)	SA IE (*n* = 85)	NSA IE (*n* = 238)	*p* - Value
Age, year	58.5 ± 16.1	58.1 ± 15.9	58.7 ± 16.2	0.776
Age				0.968
≤ 60	164 (50.8)	43 (50.6)	121 (50.8)	
> 60	159 (49.2)	42 (49.4)	117 (49.2)	
Male, n (%)	213 (65.9)	52 (61.2)	161 (67.7)	0.280
Race, n (%)				0.724
Caucasian	182 (56.4)	45 (52.9)	137 (57.6)	
African American	27 (8.4)	7 (8.2)	20 (8.4)	
Others	114 (35.3)	33 (38.8)	81 (34.0)	
BMI, kg/m^2^	26.9 ± 5.7	27.6 ± 5.9	26.7 ± 5.6	0.210
Obese (BMI > 25 kg/m^2^), n (%)	185 (61.7)	55 (69.6)	130 (58.8)	0.108
*Co-morbidities*				
Active tobacco use, n (%)	30 (9.6)	10 (12.2)	20 (8.6)	0.344
Hypertension, n (%)	199 (61.8)	54 (63.5)	145 (61.2)	0.702
Diabetes, n (%)	74 (23.0)	22 (25.9)	52 (21.9)	0.459
IV drug use, n (%)	16 (5.0)	9 (10.6)	7 (2.9)	0.005
Previous cardiac surgery, n (%)	149 (46.3)	38 (44.7)	105 (44.3)	0.949
Valve replacement in the past 5 years (%)	75 (23.2)	25 (29.4)	50 (21.0)	0.154
History of CHF, n (%)	149 (46.3)	38 (44.7)	111 (46.8)	0.735
Previous CVA, n (%)	88 (27.3)	30 (35.3)	58 (24.5)	0.055
ESRD, n (%)	37 (11.5)	14 (16.5)	23 (9.7)	0.093
Chronic lung disease, n (%)	32 (9.9)	10 (11.8)	22 (9.3)	0.512
Arrhythmia, n (%)	107 (33.2)	35 (41.2)	72 (30.4)	0.070

Values are means ± SD, or counts (%). *BMI* body mass index, *MI* myocardial infarction, *NYHA* New York Heart Association, *eGFR* estimated glomerular filtration rate

## Results

### Baseline characteristics and Causative organisms

Patient demographics are presented in Table 1. Mean age was 58.5 ± 16.1 years, 66% of patients were male, 56% were Caucasian, and mean body mass index was 26.9 ± 5.7 kg/m^2^. SA and NSA groups did not differ in baseline demographic variables. Overall, 46% of patients had a history of prior cardiac surgery, with 23% presenting with a history of valve replacement within the last 5 years. Significantly more patients in the SA group had a past medical history of intravenous drug use (10.6% SA vs. 2.9% NSA, *p* = 0.005), and there was a trend towards higher rates of prior cerebrovascular accident, end-stage renal disease, and prior arrhythmia in the SA group compared to NSA (*p* = 0.06, *p* = 0.09, and *p* = 0.07, respectively). There were no other significant inter-group differences in prior co-morbidities or surgeries.

Among the 323 IE patients, 85 (26.3%) were caused by SA; 21 patients (24.7%) had methicillin-susceptible SA (MSSA) IE; 64 patients (75.3%) had methicillin-resistant SA (MRSA) IE, and 238 (73.7%) by NSA organisms; 55 patients (23%) had coagulase-negative staphylococcus IE; 44 patients (31%) had streptococcal IE (12% viridans; 19% non-viridans); 40 patients (17%) had enterococcal IE; and 71 (51%) patients had culture-negative IE or IE caused by other uncommon microorganisms. Prosthetic valve endocarditis (PVE) was identified in 29 (34.1%) SA IE patients while 89 (33.6%) NA IE patients had PVE.

### Presenting characteristics and Echocardiographic findings

Clinical characteristics of overall patients are presented in Table 2. Significantly more patients in the SA group presented with fever (*p* = 0.02), vascular complications (major arterial emboli; septic pulmonary infarcts; mycotic aneurysm; intracranial hemorrhage; conjunctival hemorrhages; and Janeway lesions) (*p* = 0.002), septic shock (*p* = 0.04), stroke (*p* = 0.004), and embolic events (*p* = 0.02). In addition, significantly more SA patients had persistent bacteremia despite appropriate antibiotic therapy (*p* < 0.001). There were no differences in NYHA functional class, estimated glomerular filtration rate, rate of congestive heart failure, or preoperative myocardial infarct between groups. Patients in the NSA group had significantly more involvement of the aortic valve than the SA group (69% NSA vs. 49% SA, *p* = 0.001), but there was no significant difference in mitral valve involvement. Overall, 8% of patients also had concomitant tricuspid valve involvement. NSA IE patients underwent the valvular surgery significantly earlier after the hospitalization than the SA IE patients (6.92 days NSA vs. 9.94 days SA, *p* = 0.003). SA IE group had significantly longer average length of hospital stay than the patients with NSA IE (33 days SA vs. 26 days NSA, *p* = 0.046). In contrast to this trend, there is no statistical difference in the average length of ICU stay between the two groups (7.96 days SA vs. 8.00 days NSA, *p* = 0.49).

**Table 2:** Presenting Clinical Characteristics

	Overall (*n* = 323)	SA IE (*n* = 85)	NSA IE (*n* = 238)	*p -* Value
NYHA Class				0.146
Class 1 & 2	264 (81.7)	65 (76.5)	199 (83.6)	
Class 3 & 4	59 (18.3)	20 (23.5)	39 (16.4)	
Fever, n (%)	194 (60.1)	60 (70.6)	134 (56.3)	0.021
Angina, n (%)	49 (15.2)	12 (14.1)	37 (15.6)	0.753
eGFR, (ml/min)	63.3 ± 33.6	58.9 ± 34.7	64.9 ± 33.2	0.161
< 60	153 (47.4)	46 (54.1)	107 (45.0)	
≥ 60	170 (52.6)	39 (45.9)	131 (55.0)	
Vascular complications	143 (44.6)	50 (58.8)	93 (39.4)	0.002
Immunological phenomenon	65 (20.2)	19 (22.4)	46 (19.4)	0.562
New or worsening CHF	101 (31.3)	22 (25.9)	79 (33.2)	0.212
Persistent bacteremia	76 (23.6)	32 (37.7)	44 (18.6)	0.000
Leukocytosis	144 (44.6)	41 (48.2)	103 (43.3)	0.430
Pre-op septic shock, n (%)	53 (16.4)	20 (23.5)	33 (13.9)	0.039
Pre-op embolic event, n (%)	77 (24.0)	28 (33.3)	49 (20.7)	0.020
Pre-op stroke, n (%)	55 (17.0)	23 (27.1)	32 (13.5)	0.004
Pre-op MI, n (%)	29 (9.0)	8 (9.4)	21 (8.8)	0.871
Staph Aureus Species				
MRSA	21 (24.7)			
MSSA	64 (75.3)			
Prosthetic Valve Endocarditis	109 (33.7)	29 (34.1)	80 (33.6)	
Affected Valves				
Aortic valve	206 (63.8)	42 (49.4)	164 (68.9)	0.001
Mitral valve	192 (59.4)	56 (65.9)	136 (57.1)	0.159
Tricuspid valve	27 (8.4)	10 (11.8)	17 (7.1)	0.186

Values are means ± SD, or counts (%). *NYHA* New York Heart Association Class, *GFR* Glomerula Filtration Rate, *CHF* Congestive Heart Failure, *MI* Myocardial Infarction, *MRSA* Methicillin Resistant Staph Aureus, *MSSA* Methicillin Sensitive Staph Aureus

Echocardiographic variables are presented in Table 3. There was no difference in the mean ejection fraction or specific valve pathology between groups. Significantly more patients in the NSA group underwent surgery for moderate or severe aortic valve regurgitation, with no differences in grades of mitral valve regurgitation. The proportion of SA patients who had annular abscess was significantly higher compared to that of NSA group (45% SA vs. 27% NSA, *p* = 0.003), although there was no difference in the proportion of patients who had large vegetation (> 1 cm).

**Table 3:** Baseline Echocardiographic Data

	Overall (*n* = 323)	SA IE (*n* = 85)	NSA IE (*n* = 238)	*p* - Value
Left ventricular ejection fraction, %	50.9 ± 12.0	52.3 ± 11.4	50.2 ± 12.3	0.198
Severe, n (%)	52 (16.9)	10 (11.8)	42 (17.7)	0.208
Valve pathology, n (%)				
Regurgitation	2 (1.7%)	0 (0.0%)	2 (3.2%)	0.152
Stenosis	58 (48.7%)	25 (44.6%)	33 (52.4%)	0.398
Mixed	59 (49.6%)	31 (55.4%)	28 (44.4%)	0.232
Aortic regurgitation grade, n (%)				0.001
None & Mild	175 (54.2)	59 (69.4)	116 (48.7)	
Moderate & Severe	148 (45.8)	26 (30.6)	122 (51.3)	
Mitral regurgitation grade, n (%)				0.471
None & Mild	145 (44.9)	41 (48.2)	104 (43.7)	
Moderate & Severe	178 (55.1)	44 (51.8)	134 (56.3)	
Tricuspid regurgitation grade, n (%)				0.673
None & Mild	261 (80.8)	70 (82.3)	191 (80.2)	
Moderate & Severe	62 (19.2)	15 (17.7)	47 (19.8)	
Dehiscence of valve	29 (9.0)	5 (5.9)	24 (10.1)	0.245
Presence of Vegetation > 1 cm, n (%)	131 (42.1)	41 (49.4)	90 (39.5)	0.117
Presence of Annular Abscess, n (%)	103 (31.9)	38 (44.7)	65 (27.3)	0.003

### Outcomes

**Fig. 1:**
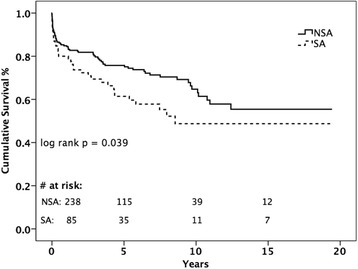
Kaplan-Meier analysis of late survival stratified by study group

Perioperative and 1-year survival, and postoperative complications are shown in Table 4. In unadjusted analyses, the overall 30-day and 1-year mortality rate of patients with SA IE were 9.4 and 20.0%, respectively, compared to 8.0 and 14.3% NSA IE (*p* = 0.68 and *p* = 0.22, respectively). However, Kaplan-Meier survival analysis (Fig. 1) of overall survival showed significantly worse survival in the SA cohort (log rank *p* = 0.039). In addition to survival, postoperative complications are also shown in Table 4. At 30-days, significantly more patients in the SA cohort experienced acute renal failure with no other differences in perioperative morbidity.

**Table 4:** Outcomes

Outcomes	Before PS Matching				After PS Matching			
	SA (*n* = 85)	NSA (*n* = 238)	OR (95% CI)	*p*-Value	SA (*n* = 64)	NSA (*n* = 64)	OR (95% CI)	*p*-Value
30-day								
Composite	40 (47.1)	91 (38.2)	1.44 (0.87–2.37)	0.160	30 (46.9)	29 (45.3)	1.06 (0.54–2.10)	0.860
Mortality	8 (9.4)	19 (8.0)	1.20 (0.50–2.85)	0.680	6 (9.4)	5 (7.8)	1.20 (0.37–3.93)	0.760
Stroke	12 (14.3)	29 (12.2)	1.20 (0.58–2.48)	0.620	9 (14.1)	9 (14.1)	1.00 (0.38–2.66)	1.000
Embolic Events	18 (21.4)	34 (14.3)	1.64 (0.87–3.09)	0.130	13 (20.3)	11 (17.2)	1.18 (0.53–2.64)	0.680
Development of ARF	24 (28.6)	43 (18.1)	1.81 (1.02–3.23)	0.043	18 (28.1)	16 (25.0)	1.22 (0.51–2.95)	0.660
1 year								
Composite	48 (56.5)	104 (43.7)	1.67 (1.01–2.75)	0.044	37 (57.8)	30 (46.8)	1.70 (0.78–3.71)	0.180
Mortality	17 (20.0)	34 (14.3)	1.50 (0.79–2.86)	0.220	13 (20.3)	9 (14.1)	1.57 (0.61–4.05)	0.350
Stroke	16 (19.1)	32 (13.5)	1.51 (0.78–2.93)	0.220	12 (18.7)	9 (14.1)	1.43 (0.54–3.75)	0.470
Embolic Events	22 (26.2)	37 (15.6)	1.93 (1.06–3.51)	0.032	16 (25.0)	13 (20.3)	1.27 (0.58–2.80)	0.550
Development of ARF	28 (33.3)	51 (21.4)	1.83 (1.06–3.18)	0.031	22 (34.4)	18 (28.1)	1.40 (0.62–3.15)	0.420

*ARF* Acute renal failure

At 1 year, the composite outcome (mortality, stroke, embolic event, or acute renal failure) occurred more frequently in SA patients (56.6% SA vs. 43.7% NSA, *p* = 0.04), which appears to be driven by higher rates of embolic events (26.2% SA vs. 15.6% NSA, *p* = 0.03) and acute renal failure (33.3% SA vs. 21.4% NSA, *p* = 0.03). Thus, while 30-day and 1-year survival were similar between groups, the SA group experienced higher rates of postoperative morbidity than the NSA group and, at late follow-up, have lower survival rates than patients with NSA IE.

Propensity score matching for the study yielded 64 matched pairs of patients who underwent valvular surgery due to SA or NSA IE. There was no significant difference in any covariate or baseline demographic among the matched pairs of patients. Among the matched pairs, the overall 30-day and 1-year mortality rate of patients with SA IE were 9.4 and 20.3%, respectively, compared to 7.8 and 14.1% NSA IE (*p* = 0.76 and *p* = 0.22, respectively). In comparison to the unadjusted analyses, the post-operative morbidity rates were similar among the matched pairs at both 30-day and 1-year.

Table 5 presents the preoperative characteristics predictive of 30-day mortality of the PS matched group. Based on the multivariable logistic regression analysis, only large vegetation was a significant predictors of 30-day mortality after the surgery (OR: 5.37, 1.90–15.2 95% CI, *p* = 0.002) while SA, nor MRSA status, or prosthetic valve endocarditis were not significant predictors of death at 30-days postoperatively (*p* = 0.68).

**Table 5:** Logistic regression analysis for preoperative characteristics predictive of 30-day mortality

	Univariable		Multivariable	
	OR (95% CI)	*p*-Value	OR (95% CI)	*p*-Value
Staph Aureus	1.20 (0.50–2.85)	0.68		
Male gender	0.87 (0.38–1.96)	0.73		
Body Mass Index	1.03 (0.96–1.10)	0.43		
Age at surgery	1.01 (0.98–1.03)	0.56		
Prior cardiac surgery	0.99 (0.45–2.20)	0.99		
Prior PCI	2.17 (0.69–6.82)	0.19	1.91 (0.54–6.77)	0.32
History of CHF	1.49 (0.68–3.29)	0.32		
Hypertension	0.90 (0.40–2.00)	0.79		
Diabetes	1.47 (0.62–3.50)	0.39		
Previous CVA	1.12 (0.47–2.65)	0.80		
MI				
> 21 days	1.48 (0.48–4.59)	0.49		
≤ 21 days	0.96 (0.12–7.72)	0.97		
Arrythmia	0.68 (0.28–1.65)	0.39		
ESRD	2.99 (1.17–7.64)	0.02	2.57 (0.58–11.47)	0.22
Chronic lung disease	0.71 (0.16–3.14)	0.65		
PAD	1.56 (0.43–5.58)	0.50		
Valve replacement within 5 years	0.94 (0.37–2.42)	0.90		
eGFR < 30	1.89 (0.79–4.54)	0.16	1.25 (0.32–4.90)	0.75
Left ventricular ejection fraction	0.97 (0.94–1.00)	0.06	0.97 (0.93–1.00)	0.05
Abscess	0.61 (0.24–1.57)	0.31		
Large vegetation (>10 mm)	5.65 (2.04–15.67)	0.001	5.37 (1.90–15.20)	0.002
New/Worsened heart failure	1.33 (0.58–3.00)	0.50		
Persistent bacteremia	1.70 (0.73–3.96)	0.22		

*PCI* Percutaneous Coronary Intervention, *CHF* Congestive Heart Failure, *CVA* Cerebrovascular Accident, *MI* Myocardial Infarction, *ESRD* End stage renal disease, *PAD* Peripheral artery disease, *GFR* Glomerular filtration rate

## Discussion

*Staphylococcus aureus* has emerged as one of the major causative organisms of IE. Studies have demonstrated that SA IE leads to an unfavorable clinical course and prognosis compared to IE caused by other bacterial organisms [4]. Our study of 323 patients with definite IE according to the modified Duke criteria demonstrate for the first time that patients with SA IE present with more severe clinical infection, as evidenced by higher rates of arrhythmia, fever, vascular complications, persistent bacteremia, annular abscess presence, septic shock, stroke, and embolic events. Mortality and morbidity rates after valve surgery were similar in patients with SA IE versus NSA IE despite the clearly increased preoperative risk, suggesting that surgery may significantly benefit SA IE patients. These novel findings may be useful to surgeons and clinicians to 1) decide if a patient represents an appropriate surgical risk, 2) predict the morbidity and mortality of a surgical intervention, and 3) potentially justify earlier operation in patients with severe SA IE.

The frequency of IE caused by SA has been reported to be increasing over recent decades [3]. SA was responsible for 26.3% of all cases of IE in the current study. Debilitating clinical scenarios such as intravenous drug use (IVDU) may be partially responsible for this trend [4]. The association between IVDU and SA IE was confirmed in our study as 10.6% of patients with IE caused by SA were active IV drug users, significantly more frequent than NSA IE patients.

Generally, SA is more aggressive than other causative organisms for IE resulting in patients presenting with more comorbidities and complications at the time of surgery. Particularly, embolic episodes and neurological complications have been well described in SA IE [5]. This virulence has been ascribed to a variety of complex factors, including its capsule and cell wall, capability to endure phagocyte’s intracellular environment, ability to attack the tissue via extracellular enzymes, and potential to acquire resistance to antibiotics [6]. Accordingly, we overall found that pre-operative development of an embolic event was significantly more frequent in SA IE patients as 33.3% of them developed embolic events compared to 20.7% of patients with IE caused by other bacterial microorganisms. Likewise, patients with SA IE were more likely to develop embolic events postoperatively (*p* = 0.020). Embolic events have a significant impact on prognosis and therefore, identifying subgroups of patients with IE who are at higher risk for embolism may be crucial for improving survival rates.

In parallel with this trend in embolic events, IE due to SA is associated with higher rates of neurological complications [5] and is the most important risk factor for all neurological complications, with an impact 2 to 3 times higher than that of other IE-causing pathogens [7]. Our study agrees with such findings, as a high frequency of pre-operative stroke (27.1% vs. 13.5%, *p* = 0.004) in overall SA versus NSA IE patients was observed. The types of stroke in our study included both ischemic and hemorrhagic stroke. It is generally suggested to delay the surgical intervention for patients with preoperative hemorrhagic stroke. In cases with hemorrhagic stroke, prognosis is worse and valve surgery is generally suggested to be postponed for at least 2–4 weeks. Conversely, if surgery is indicated after an ischemic stroke, it should not be delayed [8]. Further studies are needed to characterize the origin of stroke in SA IE patients and to evaluate whether earlier surgery for selected patients will improve the outcome of SA IE.

Previous studies have shown that SA infections are associated with septic shock of any origin [9]. Based on the study by Olmos et al. [10], SA IE patients with septic shock experienced an acute clinical onset more frequently and a more aggressive clinical course. Infection with SA leads to an increased risk for septic shock as the expression of certain virulence factors contributes to the activation of host immune system and coagulation pathways, and the pathogen replication [9, 10]. In agreement with such findings, we observed that IE caused by SA was characterized by a significantly higher incidence of preoperative septic shocks compared to NSA IE.

Prior studies have found higher mortality rates among IE patients with SA infection than among those infected with NSA. In recent large studies of endocarditis, SA has been likewise linked to a greater risk of death than other pathogens [11]. However, contrary to this general trend, our study revealed that there is no statistical significance difference in mortality in SA IE following surgery (30-day: 9.4%; 1-year: 20.3%) compared to NSA IE (30-day: 7.8%; 1-year: 14.1%, *p* = 0.22). The mortality in SA endocarditis is similar to the rates in previous studies which range from 12 to 45% [1, 12], but the present study showed that in IE, independent of risk factors, SA infection after surgical replacement does not lead to a prohibitive risk of overall mortality. Absence of difference in mortality between two groups treated surgically suggests that mortality could be effectively moderated by valvular surgery. Moreover, it is possible that the SA IE mortality would be higher if measured for a longer period of time postoperatively or if a larger sample size were studied. It is important to consider several confounding factors to the mortality outcome, including co-morbidities on presentation, site of infection, and possible disparities in patient populations. SA IE usually occurs in a debilitated clinical setting: chronic renal failure, hemodialysis, diabetes, alcoholism, cancer, immune-depression, and drug abuse [2, 4]. Our multivariate analysis also showed that SA is not an independent predictor of short and long term post-operative outcomes. Roughly equivalent surgical results between two groups despite more severe clinical presentation of SA IE patients suggests that surgery may allow convergence of the risk curves. Consequently, more detailed studies focused on identifying a relationship between SA and mortality, and on the association of valvular surgery with the improved clinical outcomes in SA IE patients are required. Finally, a full examination of all patients with SA infections who did not receive surgery is necessary to determine a complete representative risk profile of SA.

Major limitations of this study are the small number of cases and the inherent nature of the retrospective analysis. It is possible that the mortality among patients with SA IE would be higher, compared with that of NSA IE patients, if the sample size were greater. Furthermore, we cannot exclude the fact that our hospital is a tertiary referral hospital where patients with severe illnesses are referred. These patients might be a selected group with more severe complications/clinical presentation than the average population with IE. Thus, the difference in clinical characteristics and prognosis of our patients with SA IE may have been the result of referral bias. However, to our knowledge, this is the first and most detailed study to compare the clinical features and prognosis of SA IE with IE caused by other bacterial pathogens.

## Conclusion

In conclusion, this study provides data indicating that SA IE is a severe infection with worse clinical features and prognosis than IE caused by other bacterial pathogens. Our findings suggest that SA IE is associated with higher morbidity during hospitalization, such as pre-operative septic shock, embolic events, stroke and annular abscess, compared to IE caused by other bacterial organisms. Furthermore, patients with SA IE should be monitored for embolic events and acute renal failure postoperatively. Surgery may partially ameliorate the pre-operative risks of SA IE patients and early mortality from SA IE but long-term survival remains worse for patient with SA infections. Future prospective, multi-center studies are required to validate our observations and to determine the appropriate timing of surgery for patients with SA IE.

## Abbreviations

CHF: Congestive Heart Failure
HR: Hazard Ratio
IE: Infective Endocarditis
IVDU: Intravenous Drug Use
MRSA: Methicillin-Resistant *Staphylococcus aureus*
MSSA: Methicillin-Susceptible *Staphylococcus aureus*
NSA: Non-*Staphylococcus aureus*
NYHA: New York Heart Association
PS: Propensity Score
PVE: Prosthetic Valve Endocarditis
SA: *Staphylococcus aureus*
